# Correction to “Partial cooling of the upper body with a water‐cooled vest in an environment exceeding body temperature”

**DOI:** 10.1002/1348-9585.12429

**Published:** 2023-10-03

Inoue D, Nagano C, Tabuchi S, et al. Partial cooling of the upper body with a water‐cooled vest in an environment exceeding body temperature *J Occup Health*. 2023; 65: e12396.

In the “Results” section, Figure 4 was incorrect. This should have been as follows.

**FIGURE 4:**
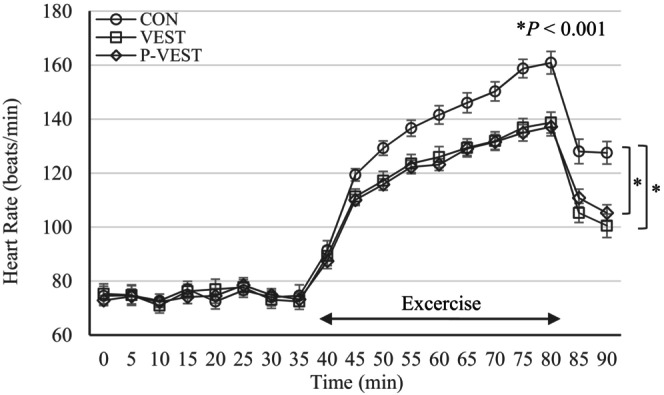
Mean heart rate trend in the CON, VEST, and P‐VEST trials. CON, group wearing long‐sleeved summer clothes; P‐VEST, group wearing clothing designed to achieve local cooling, with circulating cooled water set at 10°C; and VEST, group wearing cooling clothing with circulating cooled water set at 10°C.

We apologize for this error.

